# Abortion of Small Pox

**Published:** 1896-08

**Authors:** 


### EDITORIAL DEPARTMENT.

F. E. DANIEL, M. D., Editor.
S. E. HUDSON, M. D., Managing Editor.
A. J. SMITH. M. D., Galveston, Associate Editor.

EDITORIAL STAFF:

PROF. J. E. THOMPSON, M. D., Texas Medical College, Galveston; Surgery.
PROF. WM. KEILLER, M. D., Texas Medical College, Galveston; Obstetrics and Gynecology.
PROF. DAVID CERNA, M. D., Texas Medical College, Galveston; Therapeutics.
PROF. A. J. SMITH, M. D., Texas Medical College, Galveston; Medicine
DR. R. H. L. BIBB, City of Mexico; Foreign Correspondent.

Published Monthly at Austin, Texas, by Drs. Daniel and Hudson. Subscription price $2.00 a year in advance.

Eastern Agents: The Monihan-Fairchild Special Agency, 24 Park Place, New York City.

Official organ of the West Texas Medical Association, the Houston District Medical Association, the Austin District Medical Society, the Galveston County Medical Society, and several others.

### ABORTION OF SMALL-POX.

"HONOR TO WHOM HONOR IS DUE."—Dr. S. C. Red, editor of the *Southwestern Medical Record*, published at Houston, writes Dr. J. D. Osborn, of Cleburne, Texas, that in the July issue of his journal he had made mention of the fact that Dr. Osborn, Sr., had used bichloride mercury solution in the treatment of small-pox; and that he had received a letter from New Mexico in which the author claims priority in the use of the mercurial agent in the treatment of small-pox. Dr. Red requests Dr. Orborn to give him a statement of the facts, and Dr. Osborn refers the letter to us, with request that we protect his claim to priority. In the fulfillment of that request, we call attention to the fact that in March, 1894, Dr. T. C. Osborn wrote us that he had an idea that small-pox could be arrested in mid career, as it were, and pustulating (and pitting) prevented by the timely application (third day) of a strong germicide, his idea being that there were colonies of the germs scattered about over the surface of the body, and that they could be destroyed in situ. Accordingly, the next case of small-pox he saw, and of the diagnosis of which there was no doubt, on the third day of the fever Dr. Osborn caused the patient to be thoroughly washed in a strong solution of bichloride of mercury, using a spray of peroxide of hydrogen to the mouth and fauces at same time, and no pustules formed; and the patient recovered without a pit. The whole subject was written up by Dr. T. C. Osborn, and published in the *Texas Sanitarian* for May, 1894, giving name of patient and history of case, and treatment. In commenting on it in September, 1894, the *Sanitarian* said: "It is surprising that a claim to a discovery of such vast significance and value, given to the profession by a veteran physician of unimpeachable integrity, and of half a century observation in an extensive practice, should have attracted so little attention. If future experience should still further confirm Dr. Osborn's belief, there is no estimating the value and importance of the discovery—it will be second only to the discovery of vaccination, and will be its active coagent in the prophylaxis of this dread scourge. Dr. Osborn's name should go down to posterity, coupled with that of Jenner, as a benefactor to his race and an honor to his day and generation. * * * Had such claim been telegraphed across from Germany, and credited to some man with an unpronounceable name, the medical press of America would have been by now filled with it, and every doctor would have been reporting upon his success with the method."

We observe that in a recent issue of the *New York Medical Record*, Dr. Bryan has been experimenting with the bichloride as a preventive of pustulation, and as an abortive treatment; has gotten hold of Dr. Osborn's idea, and is laying claim to the discovery. Dr. Osborn is clearly entitled to priority in the matter.

